# Physical Activity and Negative Emotional States Among Female College Students: A Three-Wave Cross-Lagged Study

**DOI:** 10.3390/bs16091706

**Published:** 2026-09-21

**Authors:** Xinyi Chen, Wen Zhang, Chang Hu, Yuqing Yuan, Joston Gary

**Affiliations:** 1 School of Physical Education, Jiangxi Normal University, Nanchang 330022, China; 202340100801@jxnu.edu.cn (X.C.); zhangwen@jxnu.edu.cn (W.Z.); huchang@jxnu.edu.cn (C.H.); 202340100804@jxnu.edu.cn (Y.Y.); 2 School of Physical Education, Hunan First Normal University, Changsha 410205, China; 3 Department of Management and Engineering, Linköping University, SE-581 83 Linköping, Sweden; 4 Department of Economics, Management, Industrial Engineering and Tourism, University of Aveiro, 3810-193 Aveiro, Portugal; 5 School of Economics, Management and Political Science, University of Minho, 4710-057 Braga, Portugal

**Keywords:** negative emotional states, physical activity, female college students, cross-lagged analysis

## Abstract

Drawing on embodied emotion theory, this study investigated the longitudinal interplay between physical activity and negative emotional states among female college students across three waves of assessment. Female college students from five universities in Jiangxi Province, China, were assessed at three-month intervals over six months, with 1321 participants completing all three measurement waves. Physical activity was measured using the Physical Activity Rating Scale-3 (PARS-3), whereas negative emotional states were assessed using the Depression Anxiety Stress Scales-21 (DASS-21). Descriptive statistics, correlation analyses, and common method bias testing were performed using SPSS 26.0, while observed-variable and latent-variable cross-lagged panel models, together with multi-group analyses, were conducted using Mplus 8.3. Physical activity was significantly and negatively correlated with negative emotional states across all three measurement occasions (*r* = −0.363 to −0.249, all *p* < 0.01). After accounting for autoregressive effects and within-wave correlations, higher physical activity at T1 was associated with lower negative emotional states at T2 (*β* = −0.102, *p* < 0.001), and higher physical activity at T2 was associated with lower negative emotional states at T3 (*β* = −0.185, *p* < 0.001). Conversely, higher negative emotional states at T1 were associated with lower physical activity at T2 (*β* = −0.131, *p* < 0.001), and higher negative emotional states at T2 were associated with lower physical activity at T3 (*β* = −0.153, *p* < 0.001). An additional latent-variable cross-lagged panel model, in which negative emotional states were represented as a latent construct indicated by depression, anxiety, and stress, yielded the same direction and statistical significance for all four cross-lagged associations, although the standardized coefficients differed modestly in magnitude. Multi-group analyses indicated that these cross-lagged associations did not differ significantly across family residence or academic year groups. Overall, the findings point to a bidirectional pattern of longitudinal associations between physical activity and negative emotional states among female college students, in line with the embodied emotion perspective that bodily activity and emotional experiences may be interconnected over time. University mental health initiatives may therefore consider addressing both physical activity and negative emotional states, while recognizing that their longitudinal association appears to be bidirectional.

## 1. Introduction

With the popularization of higher education and increasing social competition, contemporary college students are experiencing growing psychological stress due to academic demands, employment uncertainty, and concerns about future development (Beiter et al., 2015; C. Hu et al., 2026b; Huynh et al., 2024; Jones et al., 2018; Leng et al., 2025; Z. Liu et al., 2023; Lu et al., 2024; Roy et al., 2025; Yang et al., 2025; Zou, 2026). Persistent psychological stress has been linked to a greater likelihood of experiencing negative emotional states, such as anxiety and depression, both of which represent notable challenges to college students’ psychological well-being (Deng et al., 2021; T. Huang et al., 2022; Lam et al., 2022; Li et al., 2025; Lyvers et al., 2021; Smit et al., 2023; J. Wang & Zhang, 2025). However, individuals differ in their emotional reactivity and susceptibility to stress, indicating heterogeneous patterns of negative emotional experiences (Tan et al., 2023). Among college students, female students may represent a particularly vulnerable group, as physiological characteristics, social role expectations, and differences in emotional processing may contribute to greater emotional sensitivity and higher levels of anxiety and depression compared with male peers (Bianchin & Angrilli, 2012; Farhane-Medina et al., 2022; Y. Liu et al., 2026; Nie et al., 2025; Xiang et al., 2025; Yuan et al., 2025). Persistent negative emotional states may further impair psychological adaptation, academic functioning, and social adjustment, and have been associated with adverse outcomes, including sleep disturbances, problematic internet use, substance abuse, non-suicidal self-injury, and suicidal behaviors (Gu et al., 2024; C. Hu et al., 2026b; Li et al., 2025; Rowe & Fitness, 2018; Xie et al., 2023; Young et al., 2019). Therefore, identifying modifiable behavioral factors that may help regulate negative emotional states among female college students is important.

It is noteworthy that academic time pressures, widespread screen-based media use, and prolonged sedentary behavior may collectively limit opportunities for physical activity, contributing to the high prevalence of insufficient physical activity among university students (Brown et al., 2024; Carpenter et al., 2021; Castro et al., 2020; García-Lorenzo et al., 2024; L. Xu et al., 2025). Due to factors such as body image concerns, low exercise self-efficacy, limited interest in physical activity, and time constraints, a considerable proportion of female college students do not meet the physical activity levels recommended by the World Health Organization (Aljehani et al., 2022; C. Hu et al., 2025, 2026a). A growing body of research has linked regular physical activity with better mental well-being and more adaptive psychological responses to external stressors (Balciuniene et al., 2021; Welkerling et al., 2026; G.-Y. Zhou et al., 2023). Among college populations in particular, physical activity is significantly and inversely associated with adverse psychological outcomes, including anxiety, depression, and post-traumatic stress disorder, with higher activity levels generally linked to fewer negative emotional experiences (Mu et al., 2024b). Therefore, investigating the role of physical activity in regulating emotions is of considerable practical importance for addressing negative emotional states among female college students.

Embodied emotion theory suggests that emotional experiences emerge from dynamic interactions between bodily states, action experiences, and environmental contexts rather than solely from internal cognitive processes (Seth, 2013). The body is therefore not merely a vehicle for expressing emotions but also an important foundation for shaping emotional experiences (Ye et al., 2021). From this perspective, physical activity, as a behavior that directly modifies bodily states, may contribute to emotional regulation by changing physiological and bodily experiences (Kommula et al., 2023; Remiszewski et al., 2025; Wan et al., 2025). Meanwhile, emotional states may also influence bodily behaviors by altering motivation, energy levels, and action tendencies. Negative emotional states are often accompanied by fatigue, reduced motivation, and behavioral withdrawal, which may decrease engagement in physical activity (Febrian et al., 2025; Kim et al., 2020). Reduced activity participation may further limit positive bodily experiences, potentially reinforcing negative emotional states (Herbert, 2022; X. Wang et al., 2024). Thus, embodied emotion theory provides a conceptual framework for considering how physical activity and negative emotional states may be interconnected over time. While a growing body of studies has investigated the link between physical activity and negative emotional states across different perspectives (W. Huang et al., 2025; M. Liu et al., 2024; Mu et al., 2024b; Zhao et al., 2025), with much of the existing evidence derived from cross-sectional studies that offer limited insight into the temporal sequence and potential dynamics underlying these associations. From the perspective of embodied theory, bodily states and emotional experiences are closely interconnected and mutually influential, indicating that the longitudinal association between physical activity and negative emotional states may involve reciprocal patterns rather than a single directional pathway. However, longitudinal evidence focusing specifically on female college students remains limited, particularly studies that systematically employ cross-lagged approaches to examine their reciprocal predictive relationship.

Against this backdrop, the present study, grounded in embodied theory, focused on female college students and used a longitudinal design with cross-lagged analysis to examine the temporal ordering and dynamic interplay between physical activity and negative emotional states. The study aims to deepen understanding of their longitudinal associations, clarify the direction of predictive effects, and provide both theoretical support and practical implications for promoting mental health and developing physical activity–based interventions among female college students.

Based on previous evidence and embodied emotion theory, the present study proposed the following hypotheses:

**Hypothesis** **1** **(H1).** *Physical activity would be negatively associated with negative emotional states among female college students, with higher levels of physical activity corresponding to lower levels of negative emotional states.*

**Hypothesis** **2** **(H2).** *Physical activity and negative emotional states would show reciprocal longitudinal associations across the three measurement waves. Specifically, higher levels of physical activity would be associated with lower levels of negative emotional states at the subsequent wave, whereas higher levels of negative emotional states would be associated with lower levels of physical activity at the subsequent wave.*

**Hypothesis** **3** **(H3).** *Do the reciprocal longitudinal associations between physical activity and negative emotional states differ according to family residence or academic year among female college students?*

## 2. Materials and Methods

### 2.1. Study Design and Setting

A prospective longitudinal approach was employed, involving three assessments separated by three-month intervals within a six-month observation window. This study employed a combination of stratified sampling and convenience sampling. Within the research region, stratification was conducted based on university type (comprehensive universities and normal universities) and campus environment (old campuses and new campuses). With permission from university administrative departments and under conditions suitable for on-site data collection, five universities in Jiangxi Province were selected for investigation. The target population comprised female undergraduate students in their first, second, or third academic year.

The sampling frame was restricted to first-, second-, and third-year students because the three-wave study required repeated on-campus, class-based assessments from October 2025 to April 2026. During this period, fourth-year students were primarily engaged in off-campus internships, graduation-related activities, or employment preparation, resulting in limited opportunities for consistent class-based participation across the three assessment occasions. Restricting the sampling frame to first-, second-, and third-year students therefore facilitated consistent questionnaire administration and longitudinal follow-up.

### 2.2. Participants and Procedure

Before recruitment began, the required sample size was estimated through an a priori power analysis in G*Power 3.1 (Faul et al., 2009). Because G*Power does not directly provide power calculations for a three-wave cross-lagged panel model, the principal prospective association was approximated using an F test for linear multiple regression (fixed model, R^2^ deviation from zero). Assuming a medium effect size (Cohen’s f^2^ = 0.15), an alpha value of 0.05, statistical power (1 − *β*) = 0.95, and two predictors, the analysis indicated a minimum required sample size of 107 participants. This calculation was used as a pragmatic recruitment benchmark rather than as an exact power analysis for the full structural equation model. The final longitudinal analytical sample of 1321 participants substantially exceeded this minimum. Within this predefined sampling frame, eligibility criteria included: (1) self-reported ability to participate in physical activity; (2) self-reported absence of major physical or mental health conditions; and (3) voluntary participation with signed informed consent. The survey was conducted in group formats at the class or grade level, typically during public required courses. Before completing the questionnaires, participants received an explanation of the study objectives, with emphasis placed on anonymity, voluntary participation, and data confidentiality.

**Figure 1:**
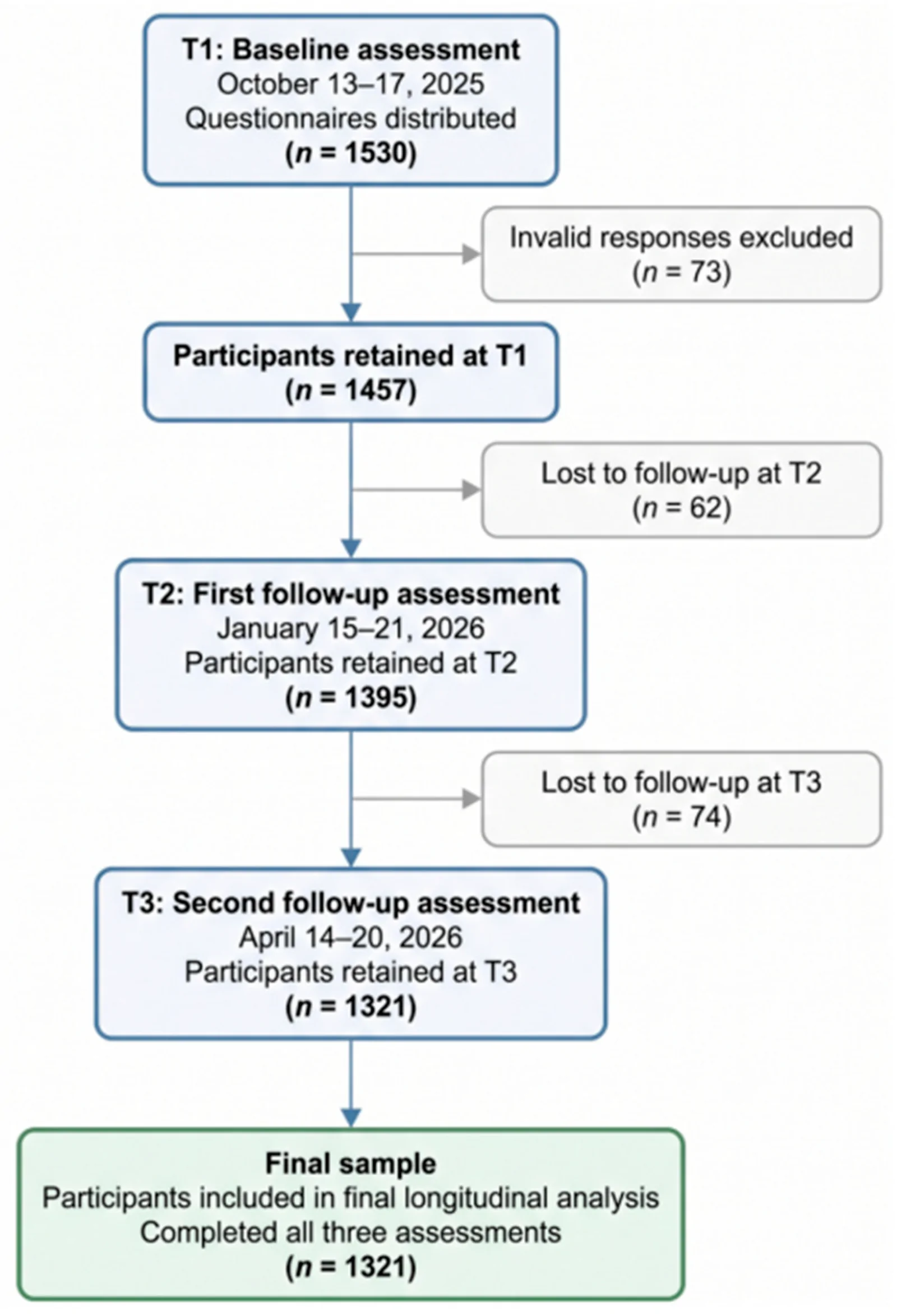
Participant recruitment and retention flow diagram.

Before data collection, all research assistants underwent training to ensure consistency in administration procedures across sites. Participants were identified using anonymous codes, allowing responses from the three assessment waves to be matched while maintaining confidentiality. Data were collected at three time points: 13–17 October 2025 (T1), 15–21 January 2026 (T2), and 14–20 April 2026 (T3). Among the 1530 questionnaires distributed at baseline, 1457 valid responses were retained. Follow-up assessments yielded 1395 valid responses at T2 and 1321 at T3. Ultimately, 1321 participants completed all three assessments, corresponding to a retention rate of 90.67%. Figure 1 presents the participant recruitment and retention process. Potential attrition bias was examined by comparing participants who completed the three-wave assessment (*n* = 1321) with those who did not complete the entire follow-up period (*n* = 136). Baseline demographic characteristics, together with the primary study variables (physical activity and negative emotional states), were evaluated between the two groups. Independent-samples t tests were conducted for continuous measures, whereas chi-square tests were applied to categorical measures. None of the examined variables differed significantly between the groups (all *p* > 0.05), indicating no evidence of substantial systematic bias associated with participant attrition.

This study was approved by the Ethics Committee of Jiangxi Normal University (IRB-JXNU-PEC-20240105). All participants voluntarily took part in the study with informed consent.

### 2.3. Measures

#### 2.3.1. Physical Activity Rating Scale (PARS-3)

The Physical Activity Rating Scale-3 (PARS-3), developed by Liang (1994), was used to assess participants’ physical activity. The scale contains three items assessing activity intensity, duration, and frequency, each rated on a five-point response scale. A total physical activity score is calculated as intensity × (duration − 1) × frequency, producing a possible score from 0 to 100. Scores of 19 or below indicate low physical activity; scores from 20 to 42 indicate moderate physical activity, and scores above 42 indicate high physical activity. The PARS-3 has been used among Chinese college students and has shown satisfactory psychometric performance in this population (C. Hu et al., 2026b; B. Huang et al., 2026; L. Xu et al., 2026). In the current sample, Cronbach’s alpha values were 0.881 at T1, 0.889 at T2, and 0.873 at T3.

#### 2.3.2. Negative Emotional States Scale

Negative emotional states were assessed using the 21-item Depression Anxiety and Stress Scale (DASS-21), originally developed by Lovibond and Lovibond (1995) and validated in Chinese college students by Gong et al. (2010). The DASS-21 comprises three correlated seven-item domains assessing depressive, anxiety, and stress symptoms. Each item is rated on a four-point scale ranging from 0 (did not apply to me at all) to 3 (applied to me very much or most of the time), with reference to experiences during the preceding week. Although these three domains are conceptually distinct, they are also closely related and share considerable variance. Evidence from higher-order and bifactor modeling has suggested the presence of a general distress component underlying responses to the DASS-21 and has indicated that the total score can be used to represent overall psychological distress or negative emotional symptoms (Alfonsson et al., 2017; Zanon et al., 2021). Similar use of the DASS-21 total score has been reported in recent research involving college students, including longitudinal studies (W. Huang et al., 2025; Yue et al., 2026). In the present study, the focus was on the overall burden of negative emotional symptoms rather than on the longitudinal trajectories of individual symptom domains. Therefore, scores across all 21 items were summed to generate a composite measure of negative emotional states, ranging from 0 to 63. Higher scores indicate greater overall negative emotional states. The composite score was treated as a continuous research measure rather than a clinical diagnostic indicator. Cronbach’s alpha values for the overall measure were 0.889 at T1, 0.880 at T2, and 0.887 at T3.

### 2.4. Bias Assessment

Potential sources of bias were also examined. Attrition bias was evaluated by comparing baseline characteristics and study variables across different levels of study retention, including participants with complete three-wave data and those who provided data only at T1. Common method bias was examined using Harman’s single-factor test because all variables were measured through self-report questionnaires.

### 2.5. Data Analysis

#### 2.5.1. Preliminary Analysis

Statistical analyses were conducted using SPSS 26.0 and Mplus 8.3. Descriptive analyses and reliability assessments were first performed, followed by Pearson correlation analyses and tests for common method bias. The possibility of common method variance was examined using Harman’s one-factor procedure (Liao et al., 2025). Longitudinal analyses were conducted among 1321 participants who completed assessments at all three time points. Participants with incomplete data across the assessment waves were excluded from the longitudinal analyses.

#### 2.5.2. Main Analysis

The longitudinal associations between physical activity and negative emotional states were examined across the three measurement occasions using cross-lagged panel models in Mplus 8.3. The primary model was an observed-variable CLPM, with physical activity operationalized using the PARS-3 total score and negative emotional states represented by the DASS-21 total score. Autoregressive effects were specified for each construct across adjacent waves, and correlations between the two constructs within each wave were freely estimated. Both measures were entered as continuous observed variables. Model estimation used robust maximum likelihood (MLR), which provides robust standard errors and fit statistics that are less sensitive to violations of multivariate normality. Standardized coefficients were reported. Only participants with complete data across the three waves were included in these longitudinal analyses (*n* = 1321).

To determine whether the longitudinal findings were sensitive to the way negative emotional states were operationalized, a second CLPM was estimated using a latent-variable approach. Physical activity remained an observed variable represented by the PARS-3 total score, whereas negative emotional states were specified as a latent factor at each measurement occasion. Depression, anxiety, and stress scores derived from the DASS-21 were used as indicators of the corresponding latent factor. The same autoregressive and cross-lagged structure was applied to the latent-variable model. This additional analysis allowed the longitudinal pattern to be examined while representing negative emotional states as a common latent construct rather than relying exclusively on the DASS-21 total score.

Overall model adequacy was assessed using the chi-square statistic, CFI, TLI, RMSEA, and SRMR, with these indices interpreted jointly in accordance with the recommendations of L. Hu and Bentler (1999). Multi-group analyses were then performed to determine whether the longitudinal associations varied by family residence or academic year. Specifically, an unconstrained model was first estimated, followed by a model imposing equality constraints on the corresponding autoregressive and cross-lagged paths. A change in CFI (ΔCFI) of no more than 0.01 was taken to indicate that imposing the equality constraints did not result in a meaningful deterioration in model fit (Cheung & Rensvold, 2002). This criterion was used to assess the similarity of the specified longitudinal paths across groups and was not taken as evidence of formal measurement or structural invariance.

## 3. Results

### 3.1. Participant Characteristics

Among the 1321 female college students included in the final analyses, 58.70% reported an urban family residence and 41.30% reported a rural family residence. Regarding academic year, 35.50%, 37.60%, and 26.90% of participants were first-year students, sophomores, and juniors, respectively. Most participants had a normal BMI (67.20%). Table 1 provides detailed demographic information, including only-child status, family monthly income, BMI categories, and academic major.

### 3.2. Assessment of Common Method Bias

All items from the physical activity and negative emotional state measures across the three time points were entered into an exploratory factor analysis. The results indicated that three, two, and three factors with eigenvalues greater than 1 were extracted at T1, T2, and T3, respectively. In the unrotated solution, the first factor accounted for 28.435%, 27.989%, and 29.276% of the total variance at T1, T2, and T3, respectively, all below the commonly accepted threshold of 40% (H. Zhou & Long, 2004). These results suggest that no substantial single-factor problem was identified. Nevertheless, the exclusive use of self-reported measures from the same participants leaves open the possibility that the observed associations were partly influenced by common method bias.

### 3.3. Descriptive Statistics and Correlation Analysis

Physical activity and negative emotional states were significantly negatively correlated at all three time points (*r* = −0.363 to −0.249, all *p* < 0.01). Regarding physical activity, significant positive correlations were observed among T1, T2, and T3 (*r* = 0.336 to 0.564, all *p* < 0.01), indicating a certain degree of temporal stability in physical activity levels. Similarly, for negative emotional states, significant positive correlations were found across T1, T2, and T3 (*r* = 0.430 to 0.597, all *p* < 0.01), suggesting a relatively high level of cross-time consistency (see Table 2). These findings provide a basis for subsequent longitudinal cross-lagged analysis.

### 3.4. Cross-Lagged Analysis of Physical Activity and Negative Emotional States

The cross-lagged panel model showed an acceptable overall fit to the observed data (χ^2^/df = 4.769, CFI = 0.993, TLI = 0.977, RMSEA = 0.053, SRMR = 0.016). Although the χ^2^/df value exceeded the commonly recommended cutoff of 3.0, model fit was evaluated using multiple indices rather than this statistic alone. The CFI, TLI, RMSEA, and SRMR values collectively indicated an adequate fit of the model to the data. In the primary observed-variable CLPM, physical activity and negative emotional states were represented by directly observed measures. Physical activity was quantified using a total score incorporating activity intensity, duration, and frequency. Negative emotional states were operationalized as a composite score derived from the DASS-21, representing participants’ overall negative emotional symptom burden. The corresponding scores at T1, T2, and T3 were entered directly into the cross-lagged model. The results indicated that both physical activity and negative emotional states exhibited good temporal stability. The autoregressive paths for physical activity were all statistically significant (T1 → T2: *β* = 0.530, *p* < 0.001; T2 → T3: *β* = 0.468, *p* < 0.001), and the autoregressive paths for negative emotional states were also significant (T1 → T2: *β* = 0.571, *p* < 0.001; T2 → T3: *β* = 0.529, *p* < 0.001).

**Figure 2:**
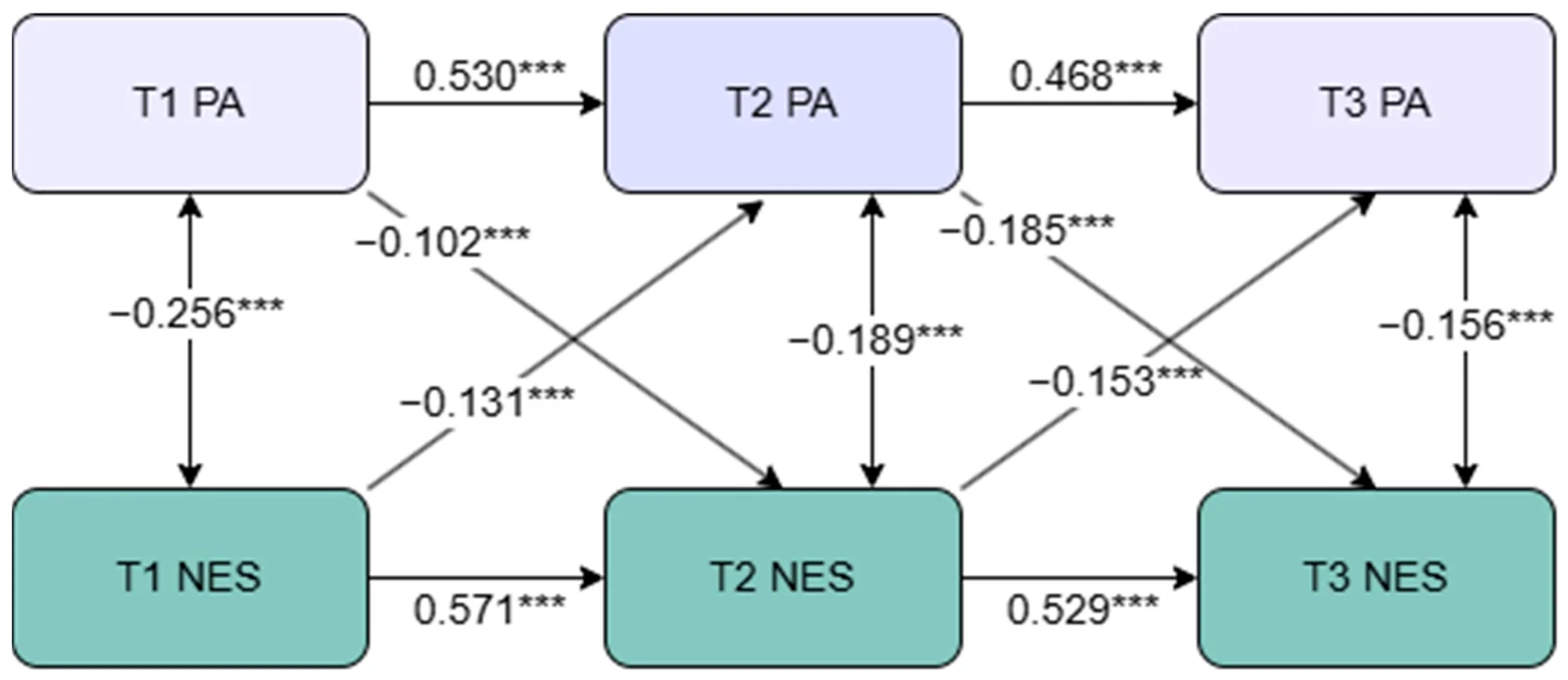
Three-wave observed-variable cross-lagged panel model of physical activity and negative emotional states among female college students. *** *p* < 0.001.

Regarding cross-lagged effects, negative emotional states at T1 significantly and negatively predicted physical activity at T2 (*β* = −0.131, *p* < 0.001), and negative emotional states at T2 significantly and negatively predicted physical activity at T3 (*β* = −0.153, *p* < 0.001). Meanwhile, physical activity at T1 significantly and negatively predicted negative emotional states at T2 (*β* = −0.102, *p* < 0.001), and physical activity at T2 significantly and negatively predicted negative emotional states at T3 (*β* = −0.185, *p* < 0.001). These findings indicated significant reciprocal longitudinal associations between physical activity and negative emotional states across the three measurement waves (Figure 2).

### 3.5. Latent Cross-Lagged Analysis of Physical Activity and Negative Emotional States

**Figure 3:**
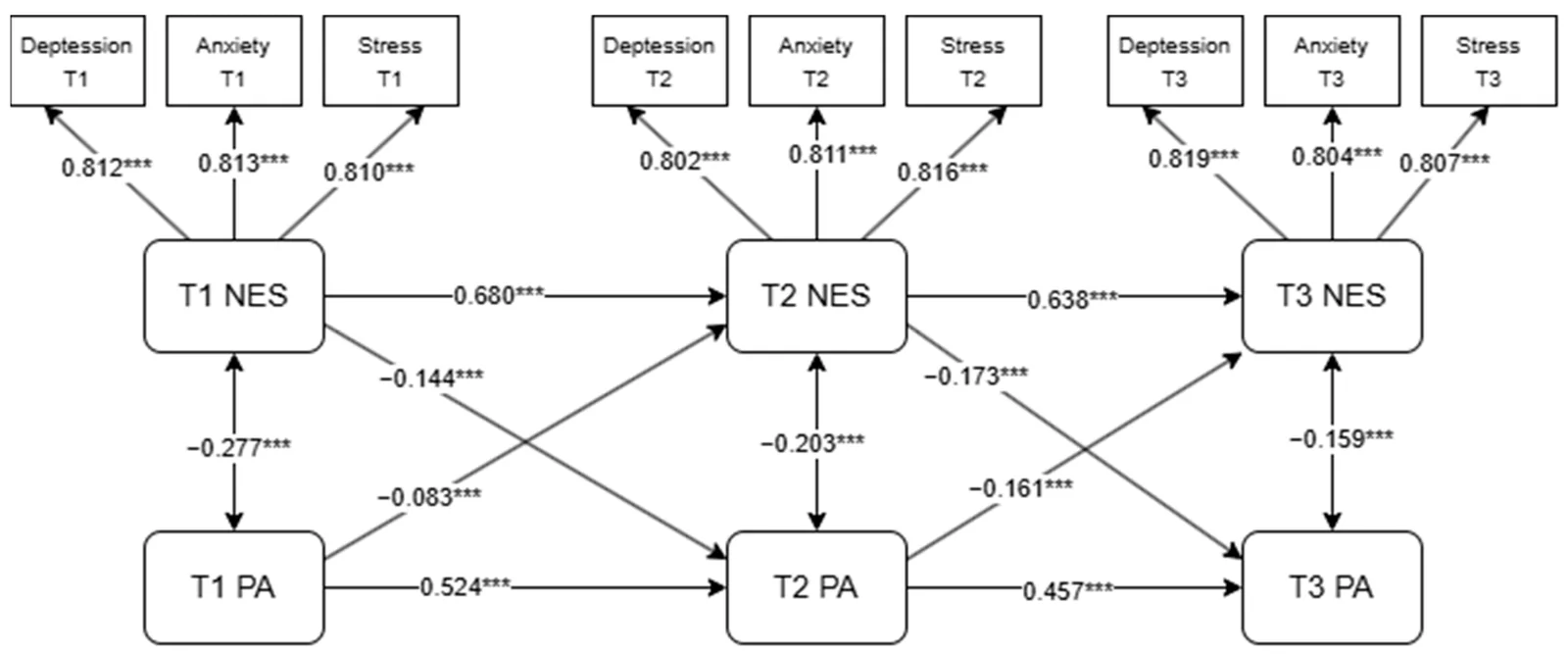
Three-wave latent cross-lagged panel model of physical activity and negative emotional states among female college students. *** *p* < 0.001.

To assess whether the longitudinal pattern was maintained when the multidimensional nature of the DASS-21 was modeled explicitly, an additional latent-variable cross-lagged panel model was conducted (Figure 3). At each measurement wave, negative emotional states were represented by a latent construct indicated by depression, anxiety, and stress scores. The standardized loadings of the three indicators were consistently high across T1, T2, and T3, ranging from 0.802 to 0.819 (all *p* < 0.001). These results supported the use of the three DASS-21 dimensions as indicators of a common latent negative emotional states construct while retaining their conceptual distinction.

The latent negative emotional states construct showed considerable temporal consistency across the three measurement occasions. Its autoregressive associations were statistically significant from T1 to T2 (*β* = 0.680, *p* < 0.001) and from T2 to T3 (*β* = 0.638, *p* < 0.001). Physical activity also showed significant temporal stability, with associations from T1 to T2 (*β* = 0.524, *p* < 0.001) and from T2 to T3 (*β* = 0.457, *p* < 0.001).

Reciprocal longitudinal associations between physical activity and latent negative emotional states were also observed. Higher negative emotional states at T1 were associated with lower physical activity at T2 (*β* = −0.144, *p* < 0.001), while higher negative emotional states at T2 were associated with lower physical activity at T3 (*β* = −0.173, *p* < 0.001). In the opposite direction, higher physical activity at T1 was associated with lower negative emotional states at T2 (*β* = −0.083, *p* < 0.001), and higher physical activity at T2 was associated with lower negative emotional states at T3 (*β* = −0.161, *p* < 0.001).

Notably, all four cross-lagged associations showed the same direction and statistical significance as those identified in the primary observed-variable model, although the standardized coefficients differed modestly in magnitude. The consistency of these patterns across the two analytical approaches provides additional support for the robustness of the longitudinal associations between physical activity and negative emotional states.

### 3.6. Multi-Group Comparisons of Cross-Lagged Paths

Multi-group cross-lagged models were conducted to assess potential differences in the longitudinal associations between physical activity and negative emotional states across family-residence groups. An unconstrained model was first estimated, allowing the corresponding autoregressive and cross-lagged paths to vary across urban- and rural-origin students. A constrained model was then estimated by imposing equality constraints on the corresponding paths across the two groups. As shown in Table 3, both models showed acceptable fit to the data, and imposing the equality constraints resulted in a minimal change in model fit (ΔCFI = 0.001). This result provided no evidence of detectable differences in the examined longitudinal paths between urban- and rural-origin students in the present sample.

Similarly, a multi-group cross-lagged model was used to examine whether the longitudinal associations differed according to academic year. An unconstrained model allowed the corresponding autoregressive and cross-lagged paths to vary across first-, second-, and third-year students. In contrast, the constrained model imposed equality constraints on these paths across the three groups. As shown in Table 3, imposing the equality constraints resulted in a minimal change in model fit (ΔCFI = 0.001). Thus, the present analysis provided no evidence of detectable differences in the examined longitudinal associations across academic-year groups.

## 4. Discussion

Grounded in embodied emotion theory, which emphasizes the interconnection between bodily states, sensorimotor experiences, and emotional processes (Barsalou, 2008; Niedenthal et al., 2005), the present three-wave study examined the longitudinal associations between physical activity and negative emotional states among female college students. The correlation analyses showed a significant inverse association between physical activity and the overall DASS-21 composite score. More importantly, the cross-lagged analyses revealed reciprocal longitudinal associations: higher physical activity at an earlier wave was associated with lower negative emotional states at the subsequent wave, whereas higher negative emotional states were associated with lower subsequent physical activity. These findings are broadly consistent with previous longitudinal and cross-lagged evidence indicating reciprocal associations between physical activity and negative emotional states (Azevedo Da Silva et al., 2012; Craig, 2002; Hou et al., 2025), as well as prospective and meta-analytic evidence linking higher physical activity with more favorable subsequent mental health outcomes (Harvey et al., 2018; Mammen & Faulkner, 2013; Pearce et al., 2022; Schuch et al., 2018). Taken together, the findings suggest that physical activity and negative emotional states are temporally interconnected across measurement occasions and are broadly compatible with embodied cognition perspectives, which view bodily activity and emotional experiences as dynamically related rather than entirely independent processes.

From an embodied perspective, emotional experiences are closely linked to bodily states, action experiences, and interactions with the surrounding environment (Critchley & Garfinkel, 2017; Seth, 2013; Wallman-Jones et al., 2021). One possible explanation for the observed association between physical activity and subsequent negative emotional states is that regular physical activity may be accompanied by physiological and interoceptive changes that are relevant to emotional regulation. Previous research has linked physical activity with alterations in stress-related physiological responses and neurobiological processes involved in emotion regulation (Kandola et al., 2019; Ye et al., 2021; Yuan et al., 2022). Physical activity may also be associated with better sleep, physical fitness, and general health, which could indirectly support more favorable emotional functioning (Jiang et al., 2025; Mu et al., 2024b). These potential mechanisms were not directly assessed in the present study, and therefore cannot be established from the current data.

Psychological and social processes may also contribute to this association. Physical activity has been associated with greater self-efficacy, particularly among college students, suggesting that perceived competence and control may represent relevant psychological correlates (Y. Hu & Liu, 2025; Mu et al., 2024a; Sheng et al., 2023; K. Wang et al., 2022). Repeated engagement in physical activity may provide opportunities for successful experiences and perceived mastery, which could support psychological adjustment to academic and daily stressors (Tang et al., 2022; K. Wang et al., 2022; R. Zhang, 2025). Physical activity may also create opportunities for social interaction and supportive relationships, potentially strengthening perceived social integration and belonging (Arango-Paternina et al., 2021; Chen et al., 2025). Social support has been identified as a relevant resource for coping with stress and emotional adjustment (Denche-Zamorano et al., 2024; Y. Wang et al., 2025). In addition, physical activity may reduce negative self-focused attention and promote emotional regulation (Li et al., 2025; Linardon et al., 2024; Y. Wang & Xia, 2026; Zhu et al., 2024). Together, these physiological, psychological, and social processes provide plausible interpretations of the observed longitudinal association, although they were not directly tested in the present study.

The observed longitudinal association between negative emotional states and subsequent physical activity is consistent with previous research showing that higher levels of anxiety, depression, and stress are associated with reduced willingness to engage in and participate in physical activity (Singh et al., 2023; Wei et al., 2024; Xiang et al., 2026). Thus, negative emotional states may represent an important psychological factor associated with subsequent physical activity engagement. From an embodied perspective, emotions are not confined to cognitive processes but are reflected in bodily sensations, action tendencies, and behavioral patterns (Seth, 2013). Negative emotional states are often accompanied by changes in bodily experiences, including increased tension, fatigue, reduced energy, and physical discomfort. Through interoceptive processes, individuals perceive and interpret internal bodily states, which may subsequently shape behavioral tendencies and emotional experiences (Seth, 2013). Accordingly, the association between negative emotional states and physical activity may involve not only cognitive appraisal but also altered perceptions of bodily states and readiness for physical activity. For example, persistent anxiety and stress may be associated with greater perceptions of fatigue or discomfort, which may reduce engagement in physical activity (Singh et al., 2023; Xiang et al., 2026; B. Xu & Amini, 2025). This possibility is also consistent with evidence that lower levels of physical activity may provide fewer opportunities for positive bodily experiences and physiological regulation, which may be related to the persistence of negative emotional states (Pelletier et al., 2017). Therefore, interoceptive processes may represent one possible theoretical explanation for the reciprocal association between physical activity and negative emotional states.

Meanwhile, depressive symptoms are frequently characterized by psychomotor retardation, reduced interest, decreased appetite, and persistent fatigue, which may be associated with a greater preference for sedentary and passive behaviors rather than active engagement (W. Huang et al., 2025; W. Zhang et al., 2025). In addition, negative emotional states may be associated with lower subsequent physical activity through reduced exercise motivation. Individuals experiencing anxiety or depression often report lower levels of self-efficacy and perceived behavioral control, which may be related to greater avoidance tendencies and reduced willingness to participate in exercise (Wanjau et al., 2023). At the same time, negative emotional states may be accompanied by increased negative cognitions and rumination, which could relate to greater psychological preoccupation with emotional distress and lower engagement in planning, initiating, and maintaining physical activity (Aljehani et al., 2022; Lu et al., 2024). For female college students, academic demands, interpersonal challenges, and career-related stress may increase vulnerability to social withdrawal and isolation. Since physical activity—particularly group-based exercise—often relies on social participation and peer support, reduced social engagement may partly contribute to the observed association between negative emotional states and subsequent levels of physical activity.

Importantly, the additional latent-variable analysis provided further support for the robustness of the findings when negative emotional states were modeled as a common latent construct. When negative emotional states were modeled as a latent construct indicated by depression, anxiety, and stress, the direction and statistical significance of all four cross-lagged associations were consistent with those observed in the original model based on the DASS-21 total score, although the standardized coefficients differed modestly in magnitude. This consistency suggests that the observed reciprocal longitudinal associations were not substantially affected by whether negative emotional states were operationalized using the DASS-21 total score or modeled as a latent construct. At the same time, depression, anxiety, and stress remain theoretically distinguishable dimensions, and the present latent-variable model does not exclude the possibility of dimension-specific longitudinal associations with physical activity. Future research could therefore examine these dimensions separately to determine whether their temporal associations with physical activity differ.

Using multi-group cross-lagged analyses, this study examined whether the longitudinal associations between physical activity and negative emotional states differed across family residence (urban vs. rural) and academic year groups. No significant differences were observed in autoregressive or cross-lagged paths between urban and rural female college students, or among first-, second-, and third-year students. The multi-group analyses did not provide evidence of detectable differences in the examined autoregressive or cross-lagged paths according to family residence or academic year. However, the absence of detectable between-group differences should not be interpreted as demonstrating strict equivalence of these associations across subgroups. Modest heterogeneity may remain undetected, and replication in larger and more socioeconomically diverse samples is warranted. Prior research has identified family background and academic stage as factors potentially related to both physical activity participation and mental health outcomes (Aceijas et al., 2017; Lema-Gómez et al., 2021; Morbée et al., 2023; D. Zhang et al., 2021). However, the absence of significant between-group differences in the present study may indicate that these demographic characteristics did not substantially alter the longitudinal associations between physical activity and negative emotional states within this sample. This pattern may partly reflect the relatively shared academic and social context of university life. Students from different family backgrounds may encounter similar study demands, daily routines, and opportunities for physical activity, leaving less room for family residence to be reflected in these outcomes. Regarding academic year, the present analyses likewise did not detect evidence that the longitudinal paths differed among first-, second-, and third-year students. Academic year was treated as a grouping variable rather than as an indicator of within-person developmental change. The academic year was treated as a grouping variable rather than a direct indicator of individual developmental change. Although the three-wave longitudinal design enabled the examination of temporal associations between physical activity and negative emotional states, comparisons across academic years involved different student cohorts rather than the same individuals followed throughout university. Therefore, these findings should not be interpreted as evidence of developmental changes across university years. Future studies employing cohort-sequential designs or longer follow-up periods are warranted to clarify further how these relationships may develop across university education.

From a practical perspective, the present findings provide practical implications for university-based mental health promotion. Given the reciprocal longitudinal associations between physical activity and negative emotional states, physical activity may be considered not only as a means of maintaining physical fitness but also as a potentially relevant factor in supporting psychological well-being. Universities may benefit from integrating structured and sustainable physical activity programs into existing mental health initiatives, with an emphasis on accessibility, regular participation, and diverse forms of engagement. Moreover, the bidirectional nature of the association suggests that interventions may address both insufficient physical activity participation and emotional difficulties associated with lower exercise engagement. Collaborative efforts between physical education departments and mental health services could facilitate the development of tailored interventions, such as supportive group-based exercise programs and activity-oriented psychological interventions, particularly for students reporting higher levels of negative emotional states. In addition, incorporating assessments of physical activity behaviors and emotional well-being into routine student health monitoring may facilitate early identification and timely support for students at risk of persistent negative emotional states. These integrated approaches may contribute to more comprehensive and sustainable strategies for promoting mental health among university students.

### Limitations

Several limitations should be considered when interpreting this study’s findings. First, the three assessments spanning six months allowed temporal patterns in the associations between physical activity and negative emotional states to be examined, including their potential bidirectional links. However, given the observational design, these findings should not be interpreted as evidence of causal relationships. Moreover, the relatively short follow-up duration may have restricted the extent to which long-term developmental patterns and dynamic changes in these variables could be captured. Future studies should adopt longer-term longitudinal designs with more measurement occasions and consider applying advanced longitudinal analytical methods, such as latent growth modeling, latent profile analysis, or latent transition analysis, to better characterize individual differences and changes over time.

Second, the participants were recruited from five universities located in Jiangxi Province, China. Although the sample included institutions with different characteristics, its concentration within a single province may limit the generalizability of the findings. Regional socioeconomic conditions, cultural norms, and lifestyle patterns may influence both physical activity behaviors and emotional experiences among college students. Therefore, the observed associations may not fully represent students from other regions with different cultural and developmental contexts. Future research should adopt broader sampling strategies, such as stratified random sampling across multiple provinces and diverse types of higher education institutions, to improve sample representativeness and external validity.

Third, reliance on self-reported measures may have introduced reporting-related biases, such as social desirability and inaccurate recall. Future studies could strengthen the assessment of these constructs by drawing on multiple sources of information and, where feasible, more objective indicators. For instance, physical activity may be evaluated using objective instruments such as accelerometers or wearable devices. Meanwhile, mental health could be assessed through physiological indicators, behavioral observations, or ecological momentary assessment, allowing for a more comprehensive and dynamic evaluation of negative emotional states.

Fourth, although the additional latent-variable analysis supported modeling negative emotional states as a common latent construct indicated by depression, anxiety, and stress, these three dimensions remain theoretically distinguishable and may exhibit dimension-specific longitudinal associations with physical activity. Because the present latent-variable model treated depression, anxiety, and stress as indicators of a common construct rather than modeling them as separate longitudinal processes, the current findings do not determine whether their individual cross-lagged associations differ. Future research could therefore examine depression, anxiety, and stress separately to further elucidate their potentially distinct associations with physical activity over time.

Finally, the present study mainly addressed the temporal interplay between physical activity and negative emotional states and further examined whether these associations varied across family residence and grade levels; however, the psychological and contextual processes underlying these relationships remain unclear. Future studies may extend this work by examining possible mediating pathways, including self-efficacy, body perception, sleep quality, and psychological resilience. Moreover, greater attention should be given to potential contextual moderators, such as exercise-related environments, campus physical activity climate, peer interactions, and family support, to better elucidate how individual and environmental factors jointly shape the relationship between physical activity and emotional well-being.

## 5. Conclusions

Using a three-wave longitudinal design and a cross-lagged panel model, this study examined the dynamic relationship between physical activity and negative emotional states in female college students. The key findings are outlined below:

(1) Physical activity was inversely associated with overall negative emotional states among female college students, with higher physical activity corresponding to a lower overall DASS-21 composite score.
(2) Physical activity and negative emotional states showed a bidirectional longitudinal association across the three measurement waves. Higher levels of physical activity were associated with lower levels of negative emotional states at subsequent waves, whereas higher levels of negative emotional states were associated with lower subsequent physical activity. This pattern was also supported by an additional latent-variable analysis in which negative emotional states were modeled as a latent construct indicated by depression, anxiety, and stress, with the direction and statistical significance of all four cross-lagged associations remaining consistent with those obtained using the DASS-21 total score.
(3) Multi-group analyses provided no evidence that the cross-lagged associations differed across family residence or academic year groups in the present sample. These findings indicate that detectable subgroup differences were not identified under the current analytical framework and should not be interpreted as evidence of strict equivalence or strong cross-group stability.

## Figures and Tables

**Table 1:** Demographic variables.

Variables	Category	Frequency	Percentage
Family residence	Urban	776	58.70%
	Rural	545	41.30%
Only child	Yes	485	36.70%
	No	836	63.30%
Academic Year	Freshman	468	35.50%
	Sophomore	497	37.60%
	Junior	356	26.90%
Family Monthly Income	≤5000	375	28.40%
	5001–10,000	506	38.30%
	10,001–20,000	278	21.00%
	>20,000	162	12.30%
BMI	Underweight	246	18.60%
	Normal	887	67.20%
	Overweight/Obese	188	14.20%
Major	Science & Engineering	369	27.90%
	Medical Sciences	427	32.30%
	Humanities & Social Sciences	186	14.20%
	Economics & Management	241	18.20%
	Education & Arts	98	7.40%

**Table 2:** Descriptive statistics of physical activity and negative emotional states indicators.

Variables	M	SD	1	2	3	4	5	6
1. T1PA	25.59	28.03	1					
2. T2PA	26.45	28.42	0.564 ***	1				
3. T3PA	25.06	27.95	0.336 ***	0.519 ***	1			
4. T1NES	16.60	10.25	−0.256 ***	−0.267 ***	−0.255 ***	1		
5. T2NES	16.20	9.83	−0.249 ***	−0.333 ***	−0.308 ***	0.597 ***	1	
6. T3NES	16.16	10.11	−0.254 ***	−0.361 ***	−0.363 ***	0.430 ***	0.590 ***	1

Note: M = means; SD = standard deviation; PA = Physical Activity, NES = Negative Emotional States; *** *p* < 0.001; The same abbreviations apply hereafter.

**Table 3:** Multi-group cross-lagged model comparisons by family residence and academic year.

Model	χ^2^	*df*	CFI	TLI	RMSEA	Absolute ΔCFI
Family Residence						
M1: Unconstrained Model	21.854	8	0.994	0.979	0.051	-
M2: Path-constrained Model	26.546	16	0.995	0.992	0.032	0.001
Academic Year						
M1: Unconstrained Model	21.652	12	0.996	0.985	0.043	-
M2: Path-constrained Model	33.761	28	0.997	0.996	0.022	0.001

Note: ΔCFI represents the absolute difference in CFI between the unconstrained and path-constrained models.

## Data Availability

The data presented in this study are available from the corresponding author upon reasonable request.

## Abbreviations

The following abbreviations are used in this manuscript:

PA	Physical Activity
NES	Negative Emotional States
PARS-3	Physical Activity Rating Scale-3
DASS-21	21-item Depression Anxiety and Stress Scale
CFI	Comparative Fit Index
TLI	Tucker–Lewis Index
RMSEA	Root Mean Square Error of Approximation
SRMR	Standardized Root Mean Square Residual
CLPM	cross-lagged panel model
MLR	Robust Maximum Likelihood
SPSS	Statistical Package for the Social Sciences
M	means
SD	standard deviation
df	degrees of freedom
